# The effect of social participation on depressive symptoms in older women adults in China: A tracking survey database

**DOI:** 10.1371/journal.pone.0322903

**Published:** 2025-05-08

**Authors:** Zicheng Jiang, Jing Deng, Yue Fan, Qianwen Song, Qi Cui, Huan Liu, Wenjun Que

**Affiliations:** 1 Organizational and Personnel Department, Jiangyin People’s Hospital Affiliated to Nantong University, Jiangyin, China; 2 College of Pharmaceutical Economics and Management, Anhui University of Traditional Chinese Medicine, Hefei, China; 3 Chengdu Shuangliu Maternal and Child Health Hospital, Chengdu, China; 4 School of Medicine and Health Management, Huazhong University of Science and Technology, Wuhan, China; 5 Ya’an Center for Disease Control and Prevention, Ya’an, China; 6 Office of the Hospital Director, The Affiliated Taizhou People’s Hospital of Nanjing Medical University, Taizhou, China; University of Sao Paulo, BRAZIL

## Abstract

Background

Under the context of active aging, older women face prominent mental health risks due to their dual vulnerability stemming from biological characteristics and social roles. Given the existing practical bottlenecks in current research namely, unclear mechanisms regarding how social participation alleviates geriatric depression and insufficient studies on population heterogeneity. It is important to analyze the impact of social participation on depressive symptoms of older women in China and to implement corresponding policy systems. Methods: Based on the 2018 health and old-age care tracking survey database in China, 3047 subjects older female were included (mean age = 69.46 years).Depressive symptoms were measured using the CESD-10 depression scale from the database, and 11 categories of social activity questionnaires were utilized to reflect the level of social participation. The impact of social participation on the depressive symptoms of older women in China was empirically analyzed using the common least squares (OLS) and two-stage least squares (2SLS) methods, and tested the endogenous and robustness of the subjects, the heterogeneity of age and urban-rural areas, and the mediating effect of self-rated health were tested using the sobel method. Results: Social participation improved the depressive symptoms of older women(β=-0.789, P < 0.01), and the variable method confirmed the estimated results through the endogeneity and robustness tests. At the same time, there was age and urban-rural heterogeneity. Social participation has a greater impact on depression symptoms in older adults 60–80 + than 80 + , and in urban areas than in rural areas. And social participation indirectly affected depression through self-assessment of health, and the indirect effect accounted for 21.4% (β = -0.139, P < 0.01). Conclusion: In the process of actively dealing with the aging of the population, the older women should be actively encouraged to participate in social activities such as community volunteering and hobby activities. According to the physical and mental development characteristics of different older groups, the government should formulate targeted social participation policies and improve relevant facilities for the elderly and the old-age security system.

## 1. Introduction

China entered the aging society in 2000, and the proportion of the older population increased significantly in the following 20 years. The aging of the population continued to deepen. The resulting health problems among the older have imposed significant pressures and serious challenges to the economic and social development of China [1]. According to the World Health Organization (WHO) statistics report in 2023, about 280 million people in the world suffer from depression, accounting for 3.8% of the world’s population, including 5% of the adult population (4% of men and 6% of women), and 5.7% of the adult population over the age of 60, with the prevalence of depression in women being about 50% higher than that in men [2]. A study on depression in the elderly aged 60 and over in China showed that the overall prevalence of depression in the older was 22.7%, among which the prevalence in older women was about 24.2% and that in older men was about 19.4%. The reality shows that paying attention to and dealing with depression in the older has become an urgent task in actively responding to population aging [3]. WHO put forward the concept of “active aging” in the late 1990s, International Classification of Functioning, Disability and Health (ICF) put forward the concept of participation in 2001and defined participation as “involvement in a life situation” or as “the lived experience” of people in the actual context in which they live [4]. And formed the policy framework of “health, participation, and protection” in 2002. It proposed that promoting the social participation of the older was an important way to actively deal with the aging of the population and improve the quality of life of the older. So far, it explains the relative authority of the definition of social participation [5]. Although the ICF has not undergone substantial revisions, WHO and the German WHO Collaborating Centre for the Family of International Classifications Research Branch for ICF have developed 34 disease-specific ICF Core Sets, optimizing their subsequent use. This advancement further underscores the dynamic nature and context-dependent characteristics of “participation” in describing functional status and conducting health assessments for specific diseases [6]. Based on the ICF’s definition of “participation” and with reference to validation studies on the validity of social participation measurement instruments in recent years [7,8]. This study defines ‘social participation’ as the dynamic process by which older individuals interact with others in the society or community through economic, cultural, political, and daily activities in their family, community, and social environments and realize their personal roles and social values. Active aging confirms the social value of the older, allowing them to return to society and participate in the economic, social, cultural and political life of the society in which they live, and giving full play to their skills, experience and wisdom can make “the social and economic pressure of aging into a driving force for sustainable development.” the all-round participation of the older in society has become the general consensus of the international community to address the aging of the population [9].

Focusing on gender differences is critical to understanding the dynamics or changes in the social dimensions of aging, especially in healthy aging program planning and policies that promote social engagement among older people [10]. Due to the different gender roles of men and women, their positions and responsibilities within the family are also different, social activity and its changes may differ between older women and men. The traditional family relationship in China is “male outside, female inside”, men are mainly engaged in activities outside the family, such as labor participation [11], personal activities are relatively free and can obtain endowment insurance accumulation, while women are mainly engaged in unpaid family activities, such as housework, raising grandchildren, etc [12]. Meanwhile, as older adults age, their cognitive abilities may decline. Depressive symptoms may further impair cognitive performance by damaging attention, executive function, and other related capacities. Leading to greater poverty and poorer health in their later years [13]. Thus, in the context of aging, the dual scarcity of economic and social capital, the health problems of older women are exacerbated. Understanding how the social participation of older women affects their depressive status will benefit older women and promote the realization of positive aging. This study explored the relationship between social participation and depression in older women, and discussed whether the differences between age and urban and rural areas were the same. Finally, we analyzed the role of self-rated health in this relationship.

## 2. Literature review and theoretical assumptions

The social participation of older people has become the focus of discussion among domestic scholars. However, there is still no unified standard for the definition of social participation [14]. Drawing on theoretical perspectives from activity theory and disability theory, researchers have discussed the impact of social participation on the mental health of older people, but there is a general consensus that social participation has a positive effect on the mental health of older people [5,15–17]. According to activity theory, older adults in a high level of social activities are more likely to experience life satisfaction and societal adaptation. They should maintain the lifestyle of the middle-aged people as long as possible, which can deny the existence of the senior citizens. Positive interactions between the older adults and the society can reduce the negative influence brought by the unfavorable situation caused by aging to a certain extent, narrow the distance between the senior citizens and the society, delay the process of brain degeneration, maintain physical and mental health, and promote the social adaptation of the senior citizens [8,18]. This theory is often used to explain the connection between social participation and mental health of older persons [19], and has undergone extensive empirical testing by scholars, all of whom support this theoretical perspective [20,21]. For example, Duanyang Gao’s (2024) study found that the higher the frequency of participation in social activities, the lower the level of depression among older adults [17].In addition, Chi Chiao’s study explored the dynamic relationship between social participation and depressive symptoms among older adults in Taiwan and found that sustained participation or the initiation of social activities in later life was significantly associated with fewer depressive symptoms [22].These findings provide strong empirical support for activity theory, suggesting that social engagement of older adults plays an important role in preventing depressive symptoms and promoting mental health.

Disability theory holds different views, the theory is proposed by E. Cumming and W. Hentry [23], its core views think that with the growth of the age, older people decline in vitality and loss of role in life. They need to be gradually freed from a variety of social obligations, to obtain a sense of autonomy, compared with the past to assume a variety of social obligations, to be able to more freely participate in their own sense of meaning or hope for their own activities, older people should play a relatively minor social role, voluntarily out of society, which will enable older people to live a quiet and satisfactory life in their later years, but also to enable social rights in an orderly manner to achieve the transfer [9,24]. In other words, older people do not need active social participation and should withdraw from their social roles in later life. Empirical research provides partial support for this theory: For example, a Korean longitudinal study of aging showed that attending religious ceremonies instead increased the risk of depressive symptoms. The positive effects of attending social gatherings were limited to older people with good mental health and had no effect on older people with pre-existing depressive symptoms [25]. Scholar Chen Chen studied that depressive symptoms play a mediating role in the relationship between social engagement and cognitive functioning, but no interaction between social engagement and depressive symptoms was observed [26].Hong mei Li’s study concluded that while social participation is a major factor in depressive symptoms in older adults, it is partially indirectly influenced through cognitive abilities [27].It should be emphasized that the theory does not advocate absolute social isolation, but rather emphasizes that older adults should strategically adjust the intensity of their participation according to their individual adaptive capacity. This dynamic and balanced perspective provides an important theoretical reference for understanding the heterogeneous analysis of older people’s social participation.

Some scholars have expanded the research content of social participation and comprehensively discussed its impact process from the perspectives of the mode, intensity, content and mechanism of social participation. The social participation patterns of older people in China can be categorized into balanced, individual-centered, family-centered and low-participation [5]. The study finds that there are significant differences between urban and rural areas in the relationship between the social participation pattern and social adaptation. Yanan Wang further studies concluded that the proportion of social activity participation in rural older adults was significantly lower than that of their urban counterparts. And He H categorized social participation into voluntary and individual types, concluding that both of them could significantly inhibit the level of depression in the older adults [21]. Other studies have determined that older adults who participate in social activities, volunteer work, and donations experience a lower risk of developing depressive symptoms, and that more frequent and diversified participation in activities further reduces the risk [28,29]. From the perspective of influence mechanism, emotional and social support [28], life satisfaction [30–32], basic psychological needs [33]; caring for grandchildren [13] and so on play a different role.

Research on women’ s mental health is also widespread [34], but less in terms of female social participation. The social participation of older women deserves more focus as improving the social structure for women of all ages is beneficial to mental health [35] Different in needs, preferences and inequalities faced by older women and men must take into account the different impacts of gender differences on social participation [36]. Additionally, the dual structure of urban and rural areas in China has a profound impact on the imbalance between urban and rural society, policy, economy and culture, and the difference between urban and rural social participation and depression is worth considering [37]. Volunteering is associated with few depressive symptoms among urban seniors, but there is no clear conclusion in rural areas [38]. Vogelsang concluded that older people living in rural areas participate less socially participation than those in urban areas and suggested that engaging in artistic or cultural activities primarily benefits the health of rural residents [39]. Thus, the relationship between social participation of older women and depressive symptoms in urban and rural areas remains unclear and warrants further exploration.

**Fig 1:**
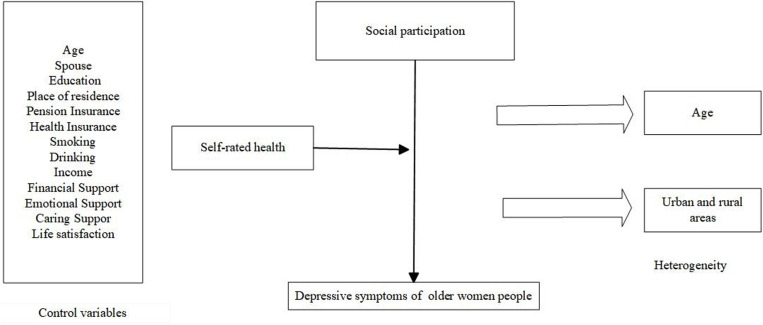
Analysis Framework of Social Participation and Depression in Older Women.

Reflecting on previous studies, while there has been a relatively comprehensive study on the impact of social participation on the depression of older people, the impact on the female group has not been focused. Current studies mainly focus on the urban older people or the overall older people as the research object. There is less consideration of differences in social participation by age group and place of residence; In addition, in the discussion of impact mechanism, the role of the intermediary variable of the aged people’s self-rated health was not considered, but rather it was taken as the control variable. Therefore, this study focused on the impact of social participation on the depressive symptoms of older women, and further analyzed the age, urban and rural differences, and the impact mechanism of self-rated health. On this basis, the paper proposes the following research hypotheses. The analysis framework of social participation and depression in older women is shown in Fig 1.

Hypothesis 1: Social participation promotes a positive promoting relationship to depression in older women.

Hypothesis 2: Social participation exhibits age heterogeneity among older women, with its impact diminishing progressively with age.

Hypothesis 3: Social participation shows urban-rural heterogeneity for older women, with a greater impact in urban areas than in rural areas.

Hypothesis 4: Social participation affects depression in older women through self-rated health assessments.

## 3. Materials and methods

### 3.1. Data sources.

Data for this article were obtained from the database of the China Health and Retirement Longitudinal Study (CHARLS) in China, Peking University in 2018. The project mainly includes high-quality microdata for households and individuals aged 45 and over,aimed at analyzing the ageing of the population in China and to promote interdisciplinary research on aging [40].

#### Sample design.

In order to ensure the unbiased and representative of the subjects, CHARLS sampling is completed through four stages, and it is sampled at the county (district)-village (neighborhood)-household-individual level. Specifically, in the county (district)-village (neighborhood) two-level sampling, CHARLS adopts the probability sampling proportional to the population size, abbreviated as PPS sampling (Probability Proportional to Size) [41]. As a first step, 150 districts and counties were randomly selected from all county units in the country, stratified by region, urban-rural, and GDP per capita according to the PPS methodology; in each county unit, three village units were randomly selected according to the PPS methodology; in each village or community, a map of the dwellings was drawn, a list of the households was produced, and a number of dwellings (the exact number of households was determined according to the age-appropriate rate and the predicted refusal rate) were randomly selected from this list; if there was more than one household of age-appropriate respondents in a dwelling, one household was randomly selected; if there was more than one household of age-appropriate respondents, one household was randomly selected as the principal respondent. If there is more than one family household of appropriate age in a residence, one household is randomly selected; if there is more than one respondent of appropriate age in a household, one person is randomly selected as the primary respondent, and the sample automatically includes the spouse of the primary respondent. At each stage of the sampling process, in order to avoid manipulation, the sampling was carried out by project staff using a computer program that did not allow substitution of samples [42]. All data collected were maintained at the Peking University Institute for Social Sciences Research and published on the CHARLS project (http://charls.pku.edu.cn) website.

#### Ethics approval.

All research was performed in accordance with relevant guidelines. All study subjects participated voluntarily and provided written informed consent, and have been performed in accordance with the Declaration of Helsinki. And was approved by the Biomedical Ethics Review Board of Peking University, China (IRB00001052–11,015).

#### Inclusion and exclusion criteria.

The subjects selected in this paper are older people female population aged 60 or above. After removing the subjects with missing values of the variables of interest in this paper, the final effective sample size was 3047.

### 3.2. Variable selection

#### 3.2.1. Dependent variable.

The dependent variable in this study is depressive symptoms. The 10-item version of the Center for Epidemiological Studies Depression Scale(CESD-10) scale is widely used in the assessment of depressive symptoms to measure the mental health of older adults, demonstrating good reliability and validity [43]. A multisite survey of persons 65 and older showed that it has the same symptom dimensions as the original CES-D with sacrifice little precision [44]. According to the answers of questionnaire CESD-10, the choices of each question were as follows: “little or no, < 1 day”; “Not too much, 1-2 days”; “Sometimes or half the time, 3-4 days”; “most of the time, 5-7 days.” Respectively assigned as’ 0, 1, 2, 3’. In the questionnaire, if any interviewee fails to answer or refuses to answer several questions, a value of “0” is assigned. It should be noted that “I am full of hope for the future” and “I am very happy” in the questionnaire are positive and require reverse coding. A higher score indicates a higher depressive mood, and the score range was 0–30 [41]. The total Cronbach’s α coefficient was 0.815 with good reliability and validity [45].

#### 3.2.2. Independent variable.

The independent variable of this article is social participation [46]. The questionnaire was selected to ask the respondents whether they had “visiting friends, associating with friends, playing cards or mahjong, social activities, and going out for tourism” and other 11 activities. To participate in any of the above social activities, they were considered to have social participation experience,which was coded as 1; absence of such participation was coded as 0.

#### 3.2.3. Control variable.

Reference to other scholars’ studies, the control of this article includes personal level, family level, social level three kinds [41,47,48]. The specific variable definitions and variable assignments are shown in Table 1.

**Table 1.** variable types, variable definitions, and variable assignments.

variable name	Variable type	Variable definition
**independent variable**		
Social participation	binary variable	Participation in any of the above social activities is considered as having social participation experience and assigned to 1; otherwise, it is considered as having no social participation experience and assigned to 0. (No = 0, Yes = 1)
**dependent variable**		
Depressive symptoms	continuously	Little or no, < 1 day = 0 (including not knowing, refusing to answer); Not too much, 1–2 days = 1; Sometimes or half the time, 3–4 days = 2; Most of the time, 5–7 days = 3 (<1 day = 0; 1–2 days = 1; 3–4 days = 2;, 5–7 days = 3)
**Control variable**		
Age	continuously	The year of questionnaire minus the year of birth gives the age, where 60–70 = 1; 70–80 = 2; Above 80 = 3.
Marital status	binary variable	No spouse (including divorced, widowed, and never married) = 0; Spouse (including married living with a spouse, married but not temporarily living with a spouse, separated) =1 (No spouse = 0; with spouse = 1)
Educational level	orderly	Illiteracy = 1, i.e., no education; Primary school and below (including unfinished primary school, private school graduation, primary school graduation) = 2; Junior high school or high school (including junior high school graduation, high school graduation, technical secondary school (including secondary normal, vocational high school graduation) = 3; College and above = 4. (Illiterate = 1; Elementary school and below = 2; Middle or high school = 3; College and above = 4.)
Place of residence	binary variable	Rural = 0; Urban and rural = 1
Endowment insurance	binary variable	According to the “Are you receiving, or are you expecting to receive or paying the following residents’ pension insurance in the future, including urban and rural residents’ pension insurance, new rural pension insurance and urban residents’ pension insurance?” Not participating = 0; Participation = 1. (Not involved = 0; involved = 1.)
Medical insurance	binary variable	According to the “Are you currently enrolled in 10 health insurance policies?” Not participating in any coverage type = 0; Participate in either = 1. (Not involved = 0; involved = 1.)
Smoke	binary variable	None = 0, have = 1
Drink wine/alcohol	binary variable	None = 0, have = 1
Total household income	continuously	Adding up all property income of the family; logarithmic processing
Financial support	binary variable	Amount of food support and cash support received by parents for all children in the past year, no financial support received = 0; Received financial support = 1. The intergenerational economic support refers to the “upward” intergenerational economic support. (No = 0; Yes = 1.)
Emotional support	binary variable	According to “How often do you see [XChildName[i]] when you and [XChildName[i]] are not living together?” Other = 0; Biannual and above = 1. The emotional support refer to “upward” emotional support. (No = 0; Yes = 1.)
Daily care	binary variable	Whether there are children living in the same family, No = 0; Yes = 1.
Life satisfaction	binary variable	According to “are you satisfied with your life? Is it extremely satisfactory, very satisfactory, relatively satisfactory, not too satisfactory or not satisfactory at all? “ Not very satisfied, not at all satisfied = 0; extremely satisfied, very satisfied, more satisfied = 1
**mediator variable**		
Self-rated health	binary variable	Good or very good = 1; General, bad or very bad = 0
**instrumental variable**		
Social relations	binary variable	If you need to take care of your daily life in the future, such as eating and dressing, do you have any relatives or friends who can take care of you for a long time?(No = 0; Yes = 1.)

#### 3.2.4. Mediator variable.

The intermediary variable in this study was self-rated health, indicating the subjective feelings of older women on their health conditions. Through the questionnaire “What do you think your health is like? Is it good, good, average, bad, or very bad? “ The answer to the. Good or very good = 1; General, bad or very bad = 0.

#### 3.2.5. Instrumental variables.

Since social participation and depression symptoms may be reverse causal [49], the baseline estimates may be biased, and the instrumental variables method is a parameter estimation method to overcome the effects of explanatory variables associated with the disturbance term. Therefore, we used the instrumental variables method to address the endogeneity problem. Refer to Liu J’s research design [50], because the older have a good social relations on the older women’s social participation behavior has an induction effect; On the other hand, the social relations of the older were not directly related to the depression of the older women. Therefore, it can be considered that the social relations of the older are in line with the requirements of instrumental variable selection. Through the questionnaire “If you need to take care of daily life in the future, such as eating and dressing, do you have relatives or friends who can take care of you for a long time?” Is measured, where “taken care of” is assigned a value of 1 and “not taken care of” is assigned a value of 0. We define this as “social relations” and use it as an instrumental variable.

### 3.3. Model setting

The dependent variable in this paper is a continuous variable, and a linear regression model is used as the baseline regression model for the study. Firstly, we analyze the influential relationship between social participation, depression symptoms and life satisfaction based on OLS model and 2SLS model. Then we discuss and analyze the endogeneity and robustness of the subjects; secondly, we analyze the age and urban-rural heterogeneity; finally, we test whether the mediating effect is significant by the sobel method and estimate the direct effect and indirect effect. In this study, the dependent variable was depressive symptoms and was a continuous variable; therefore, the linear regression model was used as the benchmark regression model, and the 2SLS regression model was used as the endogenous test. In this study, based on the selection of instrumental variables, the two-stage regression model was set as follows:


$$X_{i}=\beta_{0}+\beta_{1}Z_{1}+\Sigma \gamma D_{i}+\varepsilon_{i}$$ (1)

$$Y_{i}=\alpha_{0}+\alpha_{1}X_{i}+\Sigma \gamma D_{i}+\varepsilon_{i}$$ (2)

Equation (1) is the first-stage regression equation, in which Xi is the core explanatory variable of social participation of older women, Z1 is the instrumental variable selected in this study, and Di is the control variable of the equation; (2) The equation is the second-stage regression equation, wherein Yi is the explained variable, i.e., depression condition. β0 and α0 are intercept terms of the equation; β1, representing instrumental variable coefficient, and α1, representing core explanatory variable coefficient; γ and ε are respectively the coefficient of control variable and random error term.

First, the stepwise regression analysis was performed. Model 1 only included the core variables, that is, the relationship between social participation and depression. Model 2 added the related control variables to test the stability of the results. We selected “social relations” as the instrumental variable. and the number of the selected instrumental variables is equal to the number of the endogenous variables, and there is no over-recognition. The value of F in the model is 14.95, which is greater than the critical value of 10. It can be judged that there is no weak instrumental variable problem. Therefore, the instrumental variable meets the requirements of both correlation and exogenesis. The 2SLS model is used to ensure the reliability of the results. Model 3 represents the first-stage regression result, and Model 4 represents the second-stage regression result.

## 4. Results

A total of 3047 aged female subjects were included in this study. Personally, the proportion of the older in rural areas (74.11%) is higher than that in urban areas (25.89%), the average age is 69.46 years old, and about 58% of the subjects are aged 60–70 years old; The proportion of educated level in primary school and above was 43.55%, 56.3% of older female spouses still existed, smoking and drinking behaviors were low, and 85.4% of the older were satisfied with life evaluation. At the family level, more than 86.3% of the older women received financial assistance from their children, and more than half of them had children to take care of them. The average depression score was 10.017. The mental health status of the older women was good. The proportion of the older women with social participation (51.03%) was slightly higher than that without social participation (48.97%). However, the proportion of the older women who had a positive attitude toward self-assessment of health was only 18.8%. The pattern indicates systematic underestimation in health self-reports by Chinese older women, potentially attributable to culturally embedded health anxiety. Other descriptive statistics are presented in Table 2.

**Table 2.** Results of descriptive statistics.

Variable	N	Mean	Std. Dev.	Min	Max.
Social participation	3047	0.510	0.500	0	1
Depressive symptoms	3047	10.02	7.145	0	30
Age	3047	1.525	0.678	1	3
Spouse	3047	0.563	0.496	0	1
Education	3047	1.741	0.750	1	4
Place of residence	3047	0.259	0.438	0	1
Endowment insurance	3047	0.683	0.466	0	1
Medical insurance	3047	0.956	0.205	0	1
Smoking	3047	0.013	0.114	0	1
Drinking	3047	0.134	0.341	0	1
Income	3047	2.775	1.833	0	7.591
Financial support	3047	0.863	0.344	0	1
Emotional support	3047	0.719	0.450	0	1
Caring Support	3047	0.561	0.496	0	1
Life satisfaction	3047	0.854	0.353	0	1
Self-rated health	3047	0.188	0.391	0	1
Social relations	3047	0.681	0.466	0	1

### 4.1. Regression analysis results

The baseline regression results are presented in Table 3. Model 1 showed that social participation significantly alleviated the depression of older women at the level of 1%(β=-1.367,P < 0.01). Model 2 showed that after the relevant control variables were controlled, social participation still had a significant impact on the depression of older women(β=-0.789,P < 0.01), verifying hypothesis 1. That is, for every unit increase in social participation, the depressive symptoms of older women decreased by 0.789 units. The regression result shown in Model 3 shows that in the first stage of regression result, the social relationship of instrumental variable significantly promotes the social participation of older women at the level of 1%(β=0.052,P < 0.01). In the second stage regression result of Model 4, after considering the control endogenesis, the direction of social participation is consistent with the benchmark regression result and is at the significant level of 5%(P < 0.05), and the conclusion is robust.

**Table 3.** Baseline regression results of social participation on depression symptoms of older women in China.

	OLS		2SLS	
	Model 1	Model 2	Model 3 stage 1	Model 4 stage 2
Social participation	-1.367***	-0.789^***^		-30.582**
	(0.258)	(0.241)		(12.080)
Age		-0.371*	-0.056***	-1.995**
		(0.194)	(0.015)	(0.819)
Spouse		-0.478*	-0.054***	-2.029**
		(0.257)	(0.019)	(0.888)
Education		-0.408**	0.082***	2.067**
		(0.179)	(0.013)	(1.094)
Place of residence		-1.452***	0.041	-0.231
		(0.335)	(0.025)	(0.943)
Endowment insurance		0.515*	-0.077***	-1.715^*^
		(0.300)	(0.023)	(1.166)
Medical insurance		0.356	0.081*	2.883
		(0.582)	(0.045)	(1.822)
Smoking		0.875	0.137*	5.044
		(1.034)	(0.074)	(2.943)
Drinking		-0.381	0.089***	2.247^*^
		(0.346)	(0.026)	(1.367)
Income		0.038	-0.011**	-0.280
		(0.068)	(0.005)	(0.208)
Financial support		0.651*	0.095***	3.541^*^
		(0.364)	(0.027)	(1.459)
Emotional support		-0.625**	0.027	0.301
		(0.273)	(0.021)	(0.767)
Caring Support		-0.277	-0.022	-0.858
		(0.243)	(0.018)	(0.636)
Life satisfaction		-7.574***	0.039	-6.159^***^
		(0.335)	(0.026)	(1.003)
Social relations			0.052***	
			(0.0193)	
_cons	10.714***	18.096***	0.307***	27.565^***^
	(0.184)	(0.905)	(0.068)	(4.463)
Obs.	3047	3047	3047	3047
R-squared	0.009	0.184	0.058	0.000

Note:

*** ,

** , and

* , respectively, indicated that the estimation results were significant at the levels of 1%, 5%, and 10%, respectively;in parentheses is the standard error, the same below

### 4.2. propensity score matching

There was self-selection between social participation and depression in older women [51]. In order to solve the problem of selection bias, this paper uses propensity score matching to further estimate the relationship between the two, and uses three matching methods of neighbor matching (K = 1), radius matching and core matching to test the robustness. The results in Table 4 showed that under the three matching results, social participation could significantly alleviate the depression of older women, and the T value was significant(P < 0.05), which was consistent with the direction of the benchmark regression result coefficient and the conclusion. This indicates that the benchmark regression result also has good robustness when the sample selective bias is considered.

**Table 4:** Impacts of social participation on depressive symptoms in older women in China: propensity score matching results.

	Matching method	Average treatment effect processing effect	Standard error	T value
Social participation	K-nearest neighbor matching (k = 1)	-0.770	0.363	-2.12^**^
	Radius matching (0.05)	-0.796	0.359	-2.22^**^
	Kernel matching	-0.952	0.279	-3.42^***^

Note:

*** ,

** , and

* , respectively, indicated that the estimation results were significant at the levels of 1%, 5%, and 10%, respectively

### 4.3. Heterogeneity analysis

Traditional multiple regression analyses do not take into account differences in the likelihood of older people’s social participation, ignoring the fact that there may also be differences in the returns to social participation for older people with different levels of participation. Therefore, this paper analyzes the heterogeneity from two aspects of age and urban and rural areas, and the specific results are as follows.

#### 4.3.1. Age heterogeneity.

The age heterogeneity results are shown in Table 5. There was age heterogeneity in the influence of social participation on depression among aged women of different ages. Specifically. Social participation by older women aged 60–70 and 70–80 significantly reduced the incidence of depressive mood and promoted mental health, which was consistent with baseline results. However, the coefficient and significance of the aged women in the 70–80 age group(β=-0.633,P < 0.05) were slightly higher than those in the 60–70 age group(β=-1.258,P < 0.01), and the social participation of the aged women in the 80 + age group had no significant relationship with depression(β=-0.224,P > 0.1), which verified hypothesis 2. According to the divorced theory, the older people’s activity ability decreases and their contribution to society also decreases. People also tend to reduce the times and frequency of participating in social activities.[52]. Therefore, the 60–80 age group of older women are more willing to active and frequent social participation, and the social participation of the older with the growth of age, after 80 years of age decreased [31]Social participation had no significant effect on depressive status. Of course, this does not mean that the older women should give up social participation, but rather encourage appropriate social participation.

**Table 5.** Regression results of age heterogeneity.

	(1)	(2)	(3)
	60-70	70-80	80+
Social participation	-0.633^**^	-1.258^***^	-0.224
	(0.309)	(0.445)	(0.774)
Spouse	-0.076	-1.406^***^	1.002
	(0.335)	(0.442)	(0.953)
Education	-0.628^***^	-0.092	-0.430
	(0.224)	(0.335)	(0.653)
Place of residence	-1.486^***^	-1.889^***^	0.469
	(0.437)	(0.610)	(1.020)
Endowment insurance	0.560	0.689	-0.462
	(0.407)	(0.517)	(0.888)
Medical insurance	0.226	-0.204	1.646
	(0.906)	(0.958)	(1.309)
Smoking	0.451	1.502	3.134
	(1.395)	(2.027)	(2.407)
Drinking	-0.560	-0.763	1.600
	(0.446)	(0.630)	(1.109)
Income	0.041	-0.053	0.226
	(0.087)	(0.125)	(0.214)
Financial support	0.857^**^	0.240	-1.537
	(0.425)	(0.780)	(1.525)
Emotional support	-0.602^*^	-1.281^**^	0.429
	(0.326)	(0.551)	(1.078)
Caring Support	-0.175	-0.455	-0.112
	(0.308)	(0.447)	(0.811)
Life satisfaction	-7.902^***^	-7.377^***^	-5.721^***^
	(0.427)	(0.611)	(1.103)
_cons	17.779^***^	19.602^***^	13.180^***^
	(1.153)	(1.515)	(2.552)
Obs.	1767	960	320
R-squared	0.212	0.191	0.114

Standard errors are in parenthesis(n = 3047,60–70 = 1767; 70–80 = 960; over 80 years old = 320)

*** p < 0.01,

** p < 0.05,

* p < 0.1

#### 4.3.2. Rural-urban differences.

The results of urban-rural heterogeneity are shown in Table 6. Social participation in rural and urban areas has an impact on the depression of older women, but there is a difference in the significant level. The social participation of rural older women is only at the 10% significant level(β=-0.531,P < 0.1), This verifies hypothesis 3. while that of urban older women is at the 1% significant level(β=-1.468,P < 0.01). Specifically, for each unit of increased social participation in rural areas, the depressive status decreased by 0.531, while in urban areas it decreased by 1.468. Indicates that the rural older women’s social participation needs to be improved, This is also consistent with the findings of other scholars [39]. This may be due to the urban-rural dichotomy in Chinese society has led to a gap in resources for social activities, unequal opportunities for resource allocation and a gap in income and welfare between urban and rural older persons. Public infrastructure and social activity conditions (such as chess and card rooms, stages, fitness centers, and voluntary activity platforms) in rural areas are not as rich as those in urban areas, and their forms of social participation are single.In addition, female older persons are more involved in the care of grandchildren left behind than their urban counterparts [41],there is no spare time to engage in more social activities.

**Table 6.** Regression results of urban and rural heterogeneity.

	(1)	(2)
	rural area	urban area
Social participation	-0.531^*^	-1.468^***^
	(0.284)	(0.457)
Age	-0.646^***^	0.365
	(0.233)	(0.343)
Spouse	-0.653^**^	0.070
	(0.309)	(0.458)
Education	-0.400^*^	-0.285
	(0.226)	(0.294)
Endowment insurance	0.514	0.187
	(0.372)	(0.513)
Medical insurance	0.346	0.556
	(0.639)	(1.530)
Smoking	1.066	-1.471
	(1.119)	(2.997)
Drinking	-0.036	-1.368^**^
	(0.418)	(0.604)
Income	-0.004	0.132
	(0.087)	(0.104)
Financial support	0.810^*^	0.300
	(0.471)	(0.568)
Emotional support	-0.709^**^	-0.290
	(0.323)	(0.519)
Caring Support	-0.338	0.063
	(0.290)	(0.443)
Life satisfaction	-7.230^***^	-9.084^***^
	(0.382)	(0.712)
_cons	18.237^***^	16.435^***^
	(1.052)	(1.988)
Obs.	2258	789
R-squared	0.153	0.215

Standard errors are in parenthesis;(n = 3047;rural area = 2258; urban area = 789)

*** p < 0.01,

** p < 0.05,

* p < 0.1

### 4.4. Intermediary effect test.

**Fig 2:**
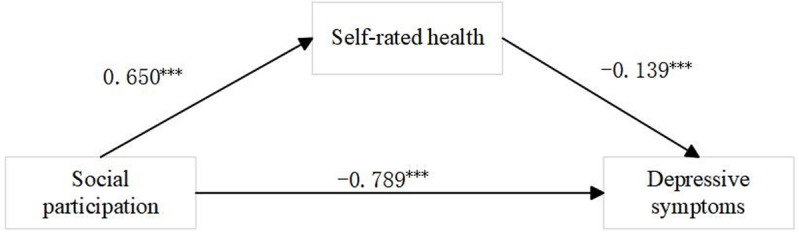
Path diagram of association between social participation and depressive symptoms. *p < 0.1, **p < 0.05, ***p < 0.01.

In order to further explore the path of social participation on the level of depression in older women, this paper examined the intermediary role of self-rated health of the older through the sobel test. The results of Table 7 show that the total effect of social participation on depression symptoms of the older women was significant (β=-0.789, P < 0.01),The direct effect of social participation on depression symptoms of the older women was statistically significant (β=-0.650, P < 0.01), with an effect share of 17.7%. In addition, the indirect effect through self-assessment of health was 0.139 or 21.4%, which was also significant (β=-0.139, P < 0.01).The results showed that social participation could improve depression and mental health of the older through self-assessment of health. Hypothesis 4 is verified. A pathway diagram of the association between social participation and depressive symptoms is shown in Fig 2.

**Table 7.** Sobel-Goodman Mediation Tests.

	effect	coefficient	P value	Effect proportion
Social participation	direct	-0.649746	<0.01	17.7%
	indirect	-0.139335	<0.01	21.4%
	Total effect	-0.789081	<0.01	

## 5. Discussion

The purpose of this study was to explore the impact of social participation on depression in older women. Specifically, Utilizing baseline regression analysis, in order to ensure the reliability of the results, we used 2SLS model and propensity score matching method(PSM) to analyze the robustness of the results from the survey data. Considering the different impacts of social participation on the depression of older women of different ages and different residence, a heterogeneous regression analysis was conducted. At the same time, the mechanism of social participation on depression in older women was studied, and the intermediary effect of self-rated health was further analyzed.

(1) First, social participation is conducive to alleviate the depression of older women and promoting mental health. This is consistent with the activity theory hypothesis, This finding supports the validity of the Activity Theory by demonstrating that individuals maintain self-identity and psychological adjustment capabilities through sustained participation in social roles and is also consistent with some scholarly studies [16,48,53]. This indicates that actively encouraging the older women to participate in social activities is conducive to improving their mental health. Specifically, due to women’s generally longer life expectancy and higher probability of living alone or being widowed, older women’s groups tend to face more severe physical and mental health risks and social support deficits during the physiological decline phase [54]. In view of this, it is recommended that at the individual level, older persons should be encouraged to take the initiative to participate in social activities such as community volunteering and hobby activities; At the national level, It should be in all aspects and areas of policy design, program operation, institutional safeguards, etc, we should pay attention to the quality of life of the older women, build a multi-level old-age care network to relieve the pressure of life care, liberate them from family labor, and enhance their social adaptation through mental health interventions [55]. Encourage the older women to participate in social and economic activities more effectively, share the fruits of social and economic development more fairly, and realize the older expectation of “having fun, having security, and doing something for the older” [56].
(2) Second, social participation of different age groups and different residence exhibits heterogeneity on the depression of older women,this supports the relevant view in the detachment theory. Notably, the social participation effect diminished in the advanced-age group (80 + years) (β = -0.224, p > 0.1), suggesting the applicability boundary of the Activity Theory: when physiological decline reduces the feasibility of role engagement, the Disengagement Theory becomes predominant. Rural elderly women exhibited a significantly weaker depressive relief effect through social participation (β = -0.531) compared to their urban counterparts (β = -1.468), highlighting that the explanatory power of the Disengagement Theory critically depends on the autonomy of disengagement: passive disengagement due to structurally deprived opportunities (e.g., inadequate rural facilities, limited leisure time) may exacerbate psychological risks. We should take into account the characteristics of physical and mental development of the older of different ages, formulate differentiated pension policies, and improve the transformation of public facilities for the older, and promote the realization of a healthy aging [37]. Moreover, 80 + Senior Women Face Aging in Life and Need the Involvement of Health Care Professionals to Provide Care Plans for Senior Patients [52]. At the same time, rural populations are often disadvantaged in terms of health access and health outcomes [57], should further reduce the difference between urban and rural resources. It also improves and enriches relevant rural services, such as increasing the number of sports equipment and providing activity areas for the elderly.so that the older in rural areas can also freely participate in a variety of social activities [58]. In addition, village councils and various organizations and agencies should promote various types of social activities, including community gatherings, volunteer services, and hobby groups, to meet the diverse interests and needs of older adults [58]. In addition, providing rural mental health support and resources such as counseling and support groups during social activities can help older adults better cope with mental health challenges such as depression [59].
(3) Finally, social participation affected the depression of older women through the intermediary variable of self-rated health. Mechanistic tests revealed a significant mediating effect of self-assessed health between social participation and depression, suggesting that mental health benefits are partially realized through the health perception enhancement pathway. This provides a mechanistic basis for the development of a joint “social participation + health management” intervention program in clinical practice - a synergistic effect can be achieved when organizing geriatric activities in conjunction with physical health interventions such as chronic disease monitoring and pain management [60]. In the context of aging, it is hoped that the results of this study will enable older women to participate more in social activities, promote the mental health of older adults, and provide a policy basis for policy makers. Therefore, to improve the level of social participation of the older, we need to improve the self-assessment of health. This requires family members to always pay attention to the physical and mental health of older women, to give family support [41]; Especially older persons in rural areas and those who lack family companionship, as their participation in social activities has a significant impact on their quality of life and should be encouraged [32]. We need the government to improve the social security system for the older, such as pension insurance, medical insurance [41]; Of course, we also call on the older themselves to develop a healthy lifestyle in the older, reduce smoking and alcohol abuse, a reasonable diet, and active exercise [57]. Finally, it will promote the individual functions of the older population and social participation, and enhance the health, well-being and quality of life of older women [61].

## 6. Limitation

There are still some limitations in this study. First, the cross-sectional study only analyzes the correlation between social participation and depressive symptoms of older adults, without clarifying causal relationship. Longitudinal data analysis is required to establish causality. The next step in our plan is to utilize the five-wave panel data from CHARLS (China Health and Retirement Longitudinal Study) to conduct more in-depth cohort studies, while incorporating cross-lagged panel models to analyze the dynamic relationships between variables.Second, although 2SLS and PSM solve the endogenous problem to some extent, this problem cannot be completely eliminate it, Although we have incorporated relevant control variables to reduce their impact on the outcomes, there remains a lack of measurement for potential confounding variables such as macro-level factors. Specifically, community environment, cultural norms, and other societal-level factors may indirectly influence mental health. These unexamined confounding effects could potentially compromise the accuracy of the mediation pathway analysis. Therefore, the potential reverse causality should be considered in further research. Third, this study only validated the overall effect of social participation, we had not analyzed whether different patterns of social participation and differences in the intensity of social participation have the same effect on the depression of older women, which should be explored in future research.

## 7. Conclusions

Based on the 2018 CHARLS data, this study used the common least squares method and instrumental variable method to empirically analyze the impact of social participation on the depressive symptoms of older women, providing evidence for improving the mental health of the older. The results of this study found that social participation of different age groups and different residence exhibited heterogeneity on the depression of older women, and the level of social participation of vulnerable groups should be improved. We found that self-rated health played an intermediary role, and more studies are still needed to explore the potential mechanism behind the relationship between social participation and depression.

## Supporting information

S1 Dataset The authors are very grateful to the China Health and Retirement Longitudinal Survey (CHARLS) for providing data and for the valuable input of all the experts who supported the study. (XLS)
